# Occurrence and Distribution of Organophosphate Flame Retardants in Tap Water System—Implications for Human Exposure from Shanghai, China

**DOI:** 10.3390/toxics12100696

**Published:** 2024-09-26

**Authors:** Yuan-Shen Zhu, Lei Zheng, Wei-Wei Zheng, Rong Zheng, Ya-Juan Wang, Bing-Qing Hu, Min-Juan Yang, Yi-Jing Zhao

**Affiliations:** 1 Center for Disease Control and Prevention, Pudong New Area, Shanghai 200136, China; 18211020127@fudan.edu.cn (Y.-S.Z.); zhengcdc@163.com (L.Z.); rzheng@pdcdc.sh.cn (R.Z.); yjwang@pdcdc.sh.cn (Y.-J.W.); bqhu@pdcdc.sh.cn (B.-Q.H.); 2 Fudan University Pudong Institute of Preventive Medicine, Shanghai 200136, China; 3 Key Laboratory of the Public Health Safety, Ministry of Education, Department of Environmental Health, School of Public Health, Fudan University, Shanghai 200032, China; weiweizheng@fudan.edu.cn; 4 Center for Water and Health, School of Public Health, Fudan University, Shanghai 200032, China

**Keywords:** organophosphate flame retardants, drinking water, daily dose, exposure assessment, risk

## Abstract

Background: The pollution of organophosphate flame retardants (OPFRs) is of global concern, but the site-specific data of OPFR concentrations in drinking water are scarce for many areas of the world outside of Europe and the US. This study aimed to investigate the occurrence and profiles of OPFRs in the tap water treatment and delivery process in Shanghai. Methods: In total, 106 samples were analyzed for 10 OPFRs, which were collected periodically from monitoring points of drinking water treatment plants and piped water between November 2021 and July 2023. The average daily doses of OPFRs through the ingestion of tap water were calculated by multiplying nominal volumes of water ingestion rates with the measured concentrations of OPFRs. Hazard quotients, the hazard index, and the carcinogenic risks of OPFRs via drinking water were used to estimate the health risks. Results: Tributyl phosphate (TBP), tris(2-chloroethyl) phosphate (TCEP), and tris (1-chloro-2-propyl) phosphate (TCIPP) were found in >90% of the tap water samples, whereas triethyl phosphate (TEP) and tris (2,3-dibromopropyl) phosphate (TDBPP) were not found in any samples. The concentrations of Σ_10_OPFRs were found at part-per-trillion ranges, with average concentrations that ranged from 86.0 ng/L in February 2023 (dry season) to 218 ng/L in July 2022 (wet season). TCIPP was the most abundant compound among the investigated OPFRs. The average daily dose of Σ_10_OPFRs via the ingestion of tap water was up to 20.4 ng/kg body weight/day. The hazard quotients of OPFRs through drinking water were in the range of 10^−5^–10^−4^, indicating low risk levels. Moreover, the hazard index of OPFRs indicated that the risk for children (2 × 10^−4^) was higher than adults (7 × 10^−5^). Conclusion: Tap water intake may be an important source of OPFRs exposure. But the risk of OPFRs for local residents is at a low level through drinking water.

## 1. Introduction

In recent decades, due to the restricted use and prohibition of some polybrominated diphenyl ether (PBDE) flame retardants, organophosphorus flame retardants (OPFRs) have become widely used as flame retardants/plasticizers and as anti-foaming agents in various commercial and industrial products [1]. OPFRs are not chemically bound to polymeric substrates and possess moderate-to-high water solubility. Therefore, these chemicals can seep from industrial materials into the surrounding environment. Although OPFRs are considered to be less pernicious than PBDE flame retardants, their increasing production and usage worldwide have led to the growing concerns of OPFR pollution.

OPFRs can enter into the aquatic environment through various pathways, including the discharge of wastewater and atmospheric deposition. The technologies currently employed in the production of drinking water are believed to have limited OPFR removal capacity [2]. Studies have reported the occurrence of OPFRs in drinking water, groundwater, surface water, and rainwater [3,4]. Stepien et al. (2013) assessed the behavior of OPFRs during riverbank filtration and groundwater flow, and they found that OPFRs were readily attenuated during bank filtration [5]. Kim U.-J. and Kannan K. (2018) found that chlorinated alkyl-OPFRs account for a similar major proportion of the total concentration in river, lake, and rainwater, but the concentrations were >3 times higher than those found in the tap water in the USA [4]. Their study also suggested that certain OPFR compounds in tap water were approximately 10 times higher in frequency than in lake and river waters.

In comparison with studies of other regions across the globe, a few studies have reported the occurrence of OPFRs in the surface water or drinking water in China [3]. Across China, the OPFR concentration in river water is generally higher than that in lake water. Alkyl-OPFRs are the predominant OPFRs in surface water samples. The concentrations of OPFRs in drinking water present an increasing trend from the inland cities of China to the coastal ones, and the concentrations were found to be significantly influenced by economic–demographic influencing factors [6]. Li et al. (2014) found that tap water contains many of the same major OPFR compounds, with concentrations that are 10–25% higher than those in bottled water [7]. Ding et al. (2015) also reported that tap water exhibited the highest exposure doses of OPFRs among various types of drinking water in eastern China [8]. Liu et al. (2019) reported that higher concentrations of OPFRs were found in the tap water in wet seasons than in the dry seasons, which indicates that there exists a seasonal variation in OPFR levels in tap water [9]. Only one study has reported the occurrence of OPFRs in the surface water of rural rivers in Shanghai. The OPFR contamination in surface river water in Shanghai was found to be at a moderate level, and these concentrations that were detected in urban rivers were significantly higher than those detected in rural rivers [10].

The quality of the source water and the tap water supply system has a great impact on the safety of water for residents. Since 2012, Shanghai city has sourced up to 70 percent of its tap water from the Qingcaosha Reservoir, which lies north of Changxing Island. The water quality in this reservoir can be at the second highest level, which means it is suitable for rare species of fish. On the other hand, the tap water system in Shanghai is in the process of being upgraded according to Shanghai Water Supply Planning (2019–2035). The coverage of deep-water treatment technologies (i.e., tertiary water treatments) in drinking water treatment plants in Pudong New Area will hit 91% in 2025. Replacements of long-distance water pipelines and water storage tanks during water delivery will be complete in 2035. In this study, we aimed to quantitatively analyze the level of OPFRs in the drinking water treatment and delivery process where water samples were collected from the inlet and outlet of drinking water treatment plants and associated residential taps in Shanghai. Furthermore, the exposure risk to OPFRs in the tap water to Shanghai residents were estimated.

## 2. Materials and Methods

### 2.1. Chemicals and Reagents

Tributyl phosphate (TBP), triethyl phosphate (TEP), tripropyl phosphate (TPrP), tris(2-chloroethyl) phosphate (TCEP), tris(1-dichloro-2-propyl) phosphate (TCIPP), triphenyl phosphate (TPhP), tris(2-butoxyethyl) phosphate (TBEP), phosphoric acid tris(1,3-dichloro-2-propyl) ester (TDCPP), and tris(2,3-dibromopropy) phosphate (TDBPP) were purchased from Alta Scientific LTD. (Tianjin, China). Tricresyl phosphate (TCP) was purchased from AccuStandard (New Haven, CT, USA). The TPhP-d_15_ purchased from Toronto Research Chemicals (North York, ON, Canada) was used as a surrogate standard. Methanol (HPLC grade) and acetonitrile (ACN, HPLC grade) were purchased from Merck KGaA (Darmstadt, Germany). Formic acid (HPLC grade) was purchased from Anaqua Chemicals Supply (Wilmington, DE, USA). Ultra-pure water (UPW, 18.2 MΩ) was produced with a Milli-Q Gradient system (Millipore, Bedford, MA, USA). Stock solutions of TPhP-d_15_ standards were prepared in acetonitrile and kept at 4 °C.

### 2.2. Sample Collection

All of the water samples, covering 60 tap water samples served by eight drinking water treatment plants and 46 plant water samples corresponding to the inlets and outlets of the drinking water treatment plants, were collected in 500 mL solvent-cleaned glass bottles with sealing films from various locations in Pudong New Area between November 2021 and July 2023. A total of 23 monitoring points of drinking water treatment plants (8) and piped water (15) were used, as shown in Figure S3. All of the samples were stored at 4 °C until analysis. The water source of all the samples came from the Qingcaosha Reservoir. Collection and preservation of water samples were performed by following the standard examination methods for drinking water [11] to reduce potential confounding factors. Details of the samples and sampling locations can be found in Text S1.

### 2.3. Analytical Methods

Each water sample was spiked with the surrogate standard and then filtered through a polyether sulfone (PES) membrane (0.22 μm, Waters, Milford, MA, USA), which was reported for the dissolved phase of the OPFRs. The filtrate was extracted using an online solid-phase extraction (SPE) method with cartridge (Sep-Pak C8, 500 mg, 3cc: Waters, Milford, MA, USA). The quantification of ten OPFRs was performed on an ultra-performance liquid chromatography tandem mass spectrometry system (AcQuity H-class, Waters, Milford, MA, USA) with a Waters BEH C18 column (2.1 mm × 50 mm, 1.7μm). The mass spectrometry was run in positive electrospray ionization mode (ESI+) with multiple reaction monitoring (MRM). Detailed information of the analysis can be found in Text S2.

### 2.4. Quality Control and Quantification

The accuracy and precision of the experiment was determined by the internal standard method. All water samples were spiked with TPHP-d_15_ at 100 ng/L as the internal standard. The standard curves for the ten OPFRs were plotted from the concentration ratios and peak area ratio of 0.0, 5.0, 10.0, 20.0, 50.0, and 100 ng/L, respectively. The linear equations and regression coefficients of the quadratic calibration curves were above 0.995 and the relative standard deviations were within 10%, which are shown in Table S1. Average recoveries of the OPFRs spiked into sample matrixes ranged from 83% to 106%, meeting the requirements of quality control.

The limits of detection (LODs) were set at a signal-to-noise (S/N) ratio of 3 (folds) and were determined to be 0.6–5.5 ng/L. The limits of quantification (LOQs) were set at an S/N ratio of 10 and were determined to be 1.9–18.2 ng/L. Details of the LODs and LOQs are shown in Table S1. Acetonitrile solvent, as a blank sample, was injected after every 10 samples to avoid contamination of the samples between injections, and no carryover of target chemicals was observed.

### 2.5. Data Handing and Analysis

The total concentrations of OPFRs (Σ_10_OPFR; the sum of the selected OPFRs) and individual OPFR compounds with a detection rate of >50% (TBP, TCIPP, and TCEP) in water samples were used for statistical analysis. The concentrations of OPFRs below the LODs were assigned a value at zero for flux calculations. The concentrations below the LOQ were substituted with a value of 1/2 LOQ. Water samples collected from the inlet and the outlet of the drinking water treatment plants, and the residential tap were defined as before-treatment plant water (b-TPW), after-treatment plant water (a-TPW), and tap water (TW), respectively. Repeated measures of ANOVA were used to indicate the variability of the concentration of OPFRs in before- and after-treatment plant water; before- and after-treatment plant water collected from the wet season and dry season; tap water and after-treatment plant water; and tap water from plastic water pipelines and metal water pipelines. Significant correlations or differences were considered when *p* < 0.05. All statistical analysis was conducted by using R 4.1.1 (R Core Team 2021, Vienna, Austria).

### 2.6. Exposure and Risk Assessment Methods

To determine whether the observed OPFR concentrations through drinking water posed a risk to human health, we conducted a preliminary exposure evaluation.

The average daily doses (ADD) of each OPFR were calculated using the following equation:

ADD=(C×IR×AP)/BW,

where C is the OPFR concentration in the tap water (ng/L), IR is the direct ingestion rate of water (L/day), AP is the absorption percent of intake (assumed to be 100%), and BW is body weight (kg). The gut absorption of OPFRs was considered to be relatively high due to their physio-chemical properties, but few studies have focused on the levels of OPFRs released from contaminated water into gastrointestinal fluids. Hence, in this exposure assessment, we assumed the absorption rate to be 100% for the purpose of a more conservative assessment and to be more in line with previous studies [4,7,8]. We also considered age- and gender-specific biometrics and exposure scenarios. Scenario 1: normal exposure with median compound concentrations and mean direct drinking water ingestion rates. Scenario 2: high exposure with mean compound concentrations and upper percentile (95%) for direct drinking water ingestion rates. Detailed information with regard to the parameters used in exposure calculation is shown in Tables S2 and S3, as obtained from the data of the Ministry of Environmental Protection of the P.R.C. (MEPC) [12,13,14].

The non-cancer and carcinogenic risks of OPFRs were estimated according to the methods recommended by US-EPA [15]. Non-cancer risks from each OPFR exposure via drinking water were appraised using the hazard quotient (HQ), which was calculated by the following equation:

HQ=ADD/RfD,

where R*f*D is the reference dose value of each OPFR (ng/kg body weight/day), as described in US-EPA (2017), by Li et al. (2019), and shown in Table S4 [16,17]. The local residents were considered to be exposed to a non-cancer risk if the value of HQ was greater than 1. The HQ of each OPFR was combined as the hazard index (HI):

HI=∑HQ.

The carcinogenic risk (CR) was assessed according to the equation:

CR=ADD×SFO,

where SFO is an oral slope factor ((ng/kg body weight/day)^−1^), representing the theoretical upper-boundary cancer potency. People are considered to be exposed to a carcinogenic risk if CR > 10^−6^. In this study, the TBP and TCEP’s CR values were estimated since their SFOs were available, as shown in Table S4 [18].

## 3. Results and Discussion

### 3.1. Concentrations of OPFRs

**Figure 1:**
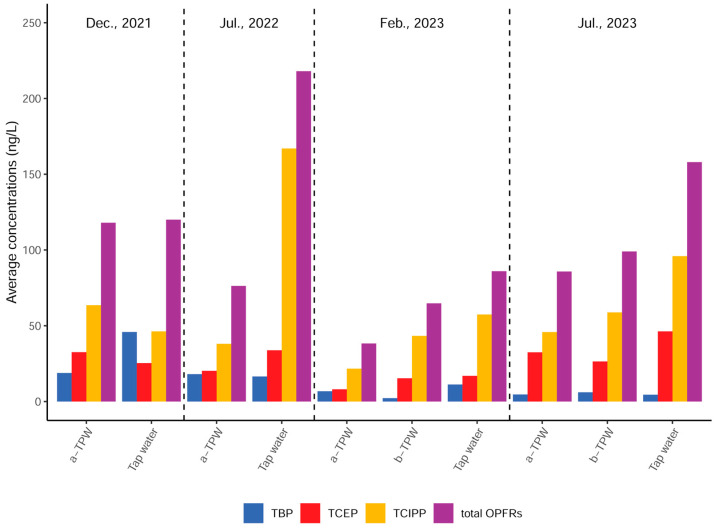
Average concentrations of the OPFRs compound in the before- and after-treatment plant water and tap water.

OPFRs were detected in all of the water samples from the tap water system of Shanghai. The total OPFR concentrations varied widely, ranging from 1.95 ng/L to 425 ng/L, with a mean value of 116 ng/L. The median concentrations of Σ_10_OPFRs in the tap water system were in the order of tap water (129 ng/L) > b-TPW (96.9 ng/L) > a-TPW (74.7 ng/L), i.e., there was a decrease of approximately 20–30%, which is shown in Figure 1.

#### 3.1.1. Concentrations of the OPFRs in the before- and after-Treatment Plant Water

The detection rates of TBP, TCIPP, and TCEP at the inlet and outlet of the drinking water treatment plants were 78%, 83%, and 80%, respectively, whereas those of the remaining OPFRs compounds were all less than 3%. The median concentrations of the three OPFRs in the latter samples (collected from February and July 2023) were 4.52 ng/L, 53.6 ng/L, and 22.7 ng/L at the inlet; and 5.96 ng/L, 34.0 ng/L, and 15.4 ng/L at the outlet of the drinking water treatment plants, representing a reduction of 19.6 ng/L and 7.3 ng/L for TCIPP and TECP, but a slight increase of 1.44 ng/L for TBP. Moreover, the median concentrations of TBP, TCIPP, and TCEP at the outlet of the drinking water treatment plants in the former samples (collected from December 2021 and July 2022) were 15.7 ng/L, 49.5 ng/L, and 22.9 ng/L, respectively. The concentrations of Σ_10_OPFRs varied widely in the before- and after-treatment plant water collected from different drinking water treatment plants. There was no significant difference between the level of OPFRs in the b-TPW and a-TPW (*p* > 0.05, partial η^2^ = 0.017), suggesting that the current drinking water plant treatment in Shanghai could not reduce the concentration of OPFRs effectively. Significant time trends in the individual or total OPFR concentrations could be found in the b-TPW and a-TPW (*p* = 0.01, partial η^2^ = 0.401), and higher concentrations were found in the wet seasons than dry seasons. This may be related to the rainfall-dependent concentration profile in Shanghai water sources.

This study area lies in the major regional and international trade zone in China, which is also where most of the country’s OPFRs are produced and consumed. More than 90% of the Shanghai residents’ raw tap water belongs to the Qingcaosha Reservoir. It is generally considered that season plays an important role for the quality of source water (b-TPW in this study). The seasonal variation in the OPFRs in surface water has been reported in several studies [4,19,20]. Higher concentrations were found in wet seasons than dry seasons [9,20]. Wet deposition fluxes with an increase through rainfall events could result in OPFRs entering the water more easily [4,21]. In addition, wet seasons are warmer than dry seasons in Shanghai. The OPFRs from consumer products and building materials could evaporate easily with an increase in atmospheric temperature [22].

Strictly speaking, the before-treatment plant water in this study was not identified with the source water. Unfortunately, it was difficult to obtain the concentration of the OPFRs in the water source in this study. The same kind of study in Zhejiang Province found lower levels of OPFRs in the source waters than in the before-treatment plant waters [18]. Similarly, in Shanghai, industrial activities around the reservoir are strictly restricted by the government to protect the water quality of the water source. The pretreatment, transportation, and store condition could introduce certain OPFRs into the early phases of the tap water system due to the relatively lesser degree of intervention and control over the long-distance water transportation from the source to the drinking water treatment plants [2]. On the other hand, traditional tap water processes have little effect on treating OPFRs, including flocculation, sedimentation, filtration, and disinfection. The removal effect of various treatment processes for different OPFRs can vary considerably, with removal rates in the conventional disposal system having been shown to be negative for TCEP and TCIPP [18]. Deep water treatment technologies (e.g., the ozonized activated carbon treatment process and its combination with the membrane treatment process) are considered effective in removing OPFRs [23]. There were two waterworks sampled using the ozonized activated carbon treatment process in this study, which showed positive removal rates (TBP: 34%, TCIPP: 31%, and TCEP: 30%). Choo (2020) and Zhang et al. (2022) reported that activated carbon filtration may be the key process for advanced drinking water treatment because it could remove approximately 50% of OPFRs [18,24]. Especially for TCEP and TCIPP, i.e., the major OPFRs in this study, it is suggested that using granular-activated carbon filtration as tertiary water treatments could dramatically reduce the OPFR levels in the tap water system of Shanghai. Further research in the improvement of removing OPFRs by advanced treatment and avoiding additional releases of OPFRs from the treatment process and in long-distance transport is needed.

#### 3.1.2. Concentrations of OPFRs in Tap Water

Similar to that which was found for the before- and after-treatment plant water samples, TBP, TCIPP, and TCEP were found in >90% of the tap water samples, whereas TEP and TDBPP were not found in any samples. The average concentrations of Σ_10_OPFRs ranged between 86.0 ng/L and 218 ng/L and were the highest in the tap water collected in July 2022 (wet season), as shown in Table 1. The concentrations of TBP and TCIPP varied widely in the tap water collected in dry seasons. What is worth further attention is the abrupt appearance of TCIPP with a high concentration in the tap water samples, which has been seldom reported previously in China. The residential tap water showed significantly higher concentrations of OPFRs than the after-treatment plant water (*p* < 0.01, partial η^2^ = 0.644). This may be related to the release of OPFRs from the water pipelines during long-distance water delivery. The distal water pipelines were mostly made of polypropylene random (PPR) (42%), followed by stainless steel (38%), polyvinyl chloride (PVC) (13%), and then ductile cast iron (7%) in this study. There was no significant increase in OPFRs in the tap water from the plastic water pipelines compared to those from metal ones (*p* > 0.05, partial η^2^ = 0.014).

There are studies that have reported the concentrations of TCIPP, TCEP, and TBP in the drinking water from various countries, including the USA, Korea, Spain, and China (Table S5). The observed concentrations of OPFRs in the tap water from Shanghai are similar to those previously reported in eastern China [6,7,8,18], but they are 2–3 times higher than those from Korea [25], Spain, and New York State in the USA [4,26]. There is a variability in the target OPFR concentrations in different studies, which could limit comparisons of the results of these studies. One study measured TCEP (median: 150 ng/L) and TCIPP (median: 220 ng/L) in the tap water from 19 drinking water treatment plants across the USA [27]. The concentrations measured in this study were 1–8 times lower than those reported earlier. Another study measured the TCEP (range: ND-48.8 ng/L) and TBP (range: ND-2.29 ng/L) in bottled water from eight brands across China [7]. Though the concentration levels of the OPFRs in bottled water were found to be lower than those found in tap water, bottle closure processes or the use of recycled PET bottles may increase the risk of OPFR contamination in certain brands of bottled water [28]. In Zhang’s study, some individual samples of household tap water showed slightly higher concentrations of OPFRs than the tap water outlet (*p* > 0.05), suggesting the probable release of OPFRs from transport pipes during water delivery [18]. From drinking water treatment plants to residential taps, the water pipelines are normally made of various materials, including certain plastics that may contain OPFRs in their composition and could have leaked through volatilization, wear, and leaching [26,29]. In China, an increasing number of families are choosing home water filters rather than bottled water to improve the taste and/or eliminate the potential harmful contaminants in tap water [30]. The effect of different types of home water filters on the OPFRs in tap water should be taken into consideration in the future.

### 3.2. Compositional Profiles of OPFR Compounds

The composition profiles of the OPFRs of the water samples from the before- and after-treatment plant water; tap water supplied directly by pipelines; tap water supplied through water storage tanks; and/or tap water were compared (Figure S4). In general, they were found to be nearly identical to the components of the OPFRs in the tap water treatment and delivery process. Among the 10 target OPFRs, TCIPP was the most predominant compound found in all the water samples, followed by TCEP and TBP. This may be related to high water solubility (TCIPP = 1.6 × 103 mg/L, TCEP = 7.0 × 103 mg/L). Significant correlations were found among TBP, TCIPP, and TCEP (Spearman’s rho = 0.204–0.714, two-tailed *p* < 0.01; Table S6). TCEP was more prone to be strongly correlated with the other two major OPFRs.

TCIPP, TCEP, and TBP are the main contaminants in the tap water system in Shanghai. The contributions of TCIPP and TCEP to the total concentrations were consistent with those in the indoor dust found across China, which together accounted for >50% [31]. This may be related to the high production volume of TCIPP and TCEP in Asia and North America. Thus far, however, no law that restricts the usage of these compounds has been implemented in Asia [32]. TCIPP and TCEP production occurs mainly in the eastern coastal areas of China such as the Yangtze River Delta [7,31]. TBP is also a key OPFR product at the initial stage of production in China due to its relatively simple production process [33]. Another source found that it is primarily bound to be inadequately treated due to the effluents from wastewater treatment plants [4,10]. The positive correlations found among the OPFRs in the types of water samples analyzed suggest similar sources of emission of OPFRs [6]. Further, correlations were found among the target OPFRs that were, in turn, attenuated with the tap water, b-TPW, and a-TPW, which could suggest that the variability of the OPFR compounds in tap water may be from the water treatment and delivery processes. Compared to the OPFRs found in the source water, these compounds in tap water could have multiple sources.

Certain OPFRs analogs, such as tris (2-ethylhexyl) phosphate (TEHP), tris (1-chloro-2-propyl) phosphate (TCPP), and triphenyl phosphate (TPP), were not investigated in this study because the concentrations of the routine detection for these OPFRs in drinking water were often significantly under the levels found in the majority of other studies. In Zhang’s study, a certain percentage (10.42%) of TEHP was found in the tap water samples [18]. TEHP is mainly used as a solvent in the production of hydrogen peroxide, which is the key chemical used in the processing of plastics and fibers [34]. Hydrogen peroxide is one of the active ingredients in silver ion disinfectants, and these disinfectants are commonly used in the regular disinfection of water supply pipes [35]. TCPP was the main OPFR found in two studies, and the mean concentrations were 20.0 ng/L and 33.4 ng/L, respectively [7,18]. TCPP, which is widely used in polyurethane flexible and rigid foams, demonstrated a relatively high water solubility. TPP was also found to be the main OPFR, with a mean value of 40.0 ng/L, in one of the studies mentioned above [7]. Another study reported positive correlations between TPP and other OPFRs (*p* < 0.01), indicating a common source [4]. Shanghai, the coastal province of China, has wide international trade, leading to a more prevalent use of various OPFRs, so further research in the pollution of emerging OPFRs is needed.

### 3.3. Exposure Assessment of the OPFRs through Tap Water Consumption

**Figure 2:**
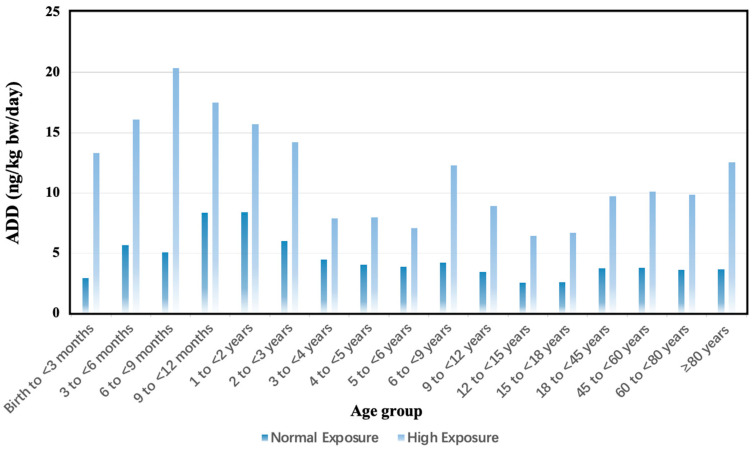
The average daily doses (ADD) of the ∑_10_OPFRs for age groups under normal- and high- exposure scenarios.

**Figure 3:**
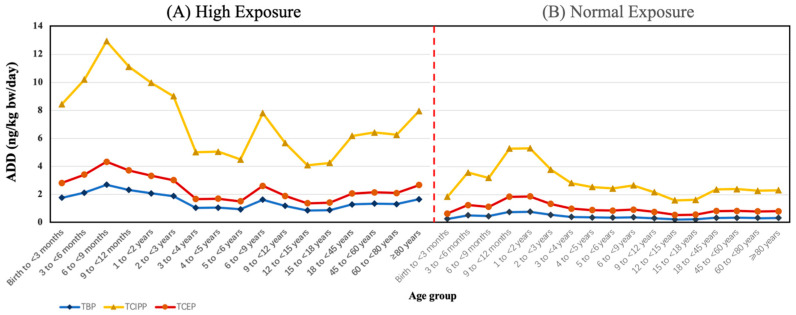
The average daily doses (ADD) of the main OPFRs for age groups under high- (**A**) and normal- (**B**) exposure scenarios.

The ADD of the OPFRs was calculated based on normal- and high-exposure scenarios (Figure 2). Under the normal-exposure scenario, the ADD of the Σ_10_OPFRs ranged between 2.53 ng/kg body weight/day and 8.42 ng/kg body weight/day. Under the high-exposure scenario, the ADD of the Σ_10_OPFRs ranged between 6.44 ng/kg body weight/day and 20.4 ng/kg body weight/day. Among the various age groups, infants were the highly exposed group. The total exposure to OPFRs was higher in males (5.15–15.8 ng/kg body weight/day) than in females (4.81–9.88 ng/kg body weight/day) due to the higher water consumption in males (Figure S5). TCIPP was the highest contributor to the ADD of the Σ_10_OPFRs in different populations, followed by TCEP and TBP (Figure 3).

The ADD of the OPFRs through tap water ingestion in Shanghai was similar to those calculated for South Korea and other regions of China [7,18,25], but it was 2–10 times higher than New York State in the USA [4]. Previous studies across China have reported that food consumption and air inhalation are the main exposure pathways to OPFRs. In comparison to the reported exposure dose of OPFRs through air inhalation from western China [36], northern China [37], and mainland China [38], the ADD via water ingestion was close to indoor dust ingestion (5 ng/kg body weight/day for adults; 6 ng/kg body weight/day for children [37]), but it was also at least double of that present in outdoor dust (0.16 ng/kg body weight/day for adults; 0.88 ng/kg body weight/day for children [38]) and air (0.41 ng/kg body weight/day for adults; 0.47 ng/kg body weight/day for children [36]). In comparison to the dietary intakes of OPFRs, intake from tap water ingestion was at least one order of magnitude lower [39,40,41]. The estimated dietary intakes of OPFRs varies between studies due to their variation in foodstuffs. Hu et al. (2014) suggested that rice was the most contaminated food sample with OPFRs in China, with a total adult exposure dose of up to 570 ng/kg body weight/day [39]. Ding et al. (2018) estimated the total OPFR exposures of 55 ng/kg body weight/day and 98 ng/kg body weight/day for adults and children, which were calculated from various food matrices involving cereals, vegetables, meat, eggs, poultry, aquatic products, tofu, and milk [40]. In another study, Zhao et al. (2019) estimated a total exposure dose of 44 ng/kg body weight/day for adults calculated across nine food categories (covering about 95% total per capita food consumption) [41]. Generally speaking, children were found to be exposed to higher doses of OPFRs compared to adults due to their lower body weight, more frequent hand-to-mouth activities, and food preference [16,42]. Overall, drinking water exposure to OPFRs was found to chiefly occur in areas close to indoor dust and air exposure but less than the degree due to food exposure.

The OPFR exposure by indirect ingestion of river/lake water during swimming was estimated in the USA, which was up to 15.8 ng/event for children and 9.28 ng/event for adults [4]. In Shanghai, the local residents always go swimming in natatoriums, in which the water comes from the tap water system. Possible indirect ingestion of tap water during swimming could be considered in future exposure calculations. The external exposure assessment is a useful tool for assessing the source or pathway of pollutants to the body; however, the total exposure of humans to OPFRs, including all routes of absorption, remains unknown. Furthermore, the influences of administrative, regional, and socioeconomic factors on exposure risks cannot be estimated well in the current models. Hence, it is recommended to establish a comprehensive exposure assessment model based on the national database.

### 3.4. Risk Assessment of OPFRs through Tap Water Consumption

**Figure 4:**
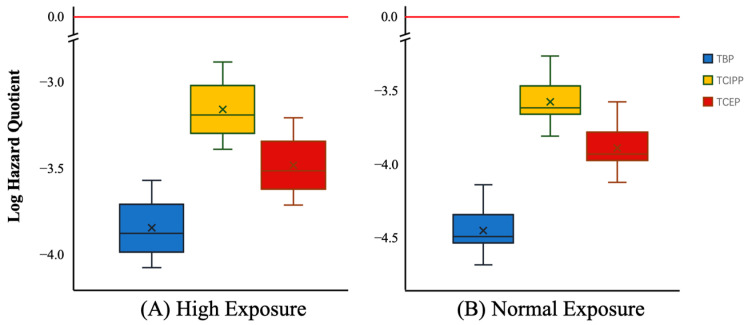
The hazard quotients (HQs) determined via tap water ingestion under high- (**A**) and normal- (**B**) exposure scenarios.

The results of the non-carcinogenic risk from the ingestion of TBP, TCIPP, and TCEP in tap water are shown in Figure 4. No risk assessment was performed for the rest of the OPFRs due to no or extremely low detection in the samples. The ADD of the OPFRs via tap water was 3 to 5 orders of magnitude different from the R*f*D for both of the normal- and high-exposure scenarios, which indicated that the non-carcinogenic risks of the three OPFRs were within acceptable limits. The HI values for ∑_10_OPFRs in tap water and the values for the high-exposure scenario (6.9 × 10^−4^–2.2 × 10^−3^) with HI < 1 were calculated, and the degree of concentration found suggested that the combined exposure to the three main OPFRs did not pose a non-carcinogenic risk to humans. The results of TBP and TCEP carcinogenic risk are shown in Table 2, with CR values ranging from 2.7 × 10^−8^ to 8.7 × 10^−8^ and 7.7 × 10^−9^ to 2.4 × 10^−8^ for the high-exposure scenario, respectively. All of the CR values were less than the threshold value of 10^−6^. The overall carcinogenic risk to local residents from the ingestion of tap water was too low to be of a concern.

The widespread existence of OPFRs in drinking water has led to concerns regarding the risks to human health in China. One study analyzed the health risk of OPFRs in drinking water covering 79 cities of China, and it was suggested that there was a potential cancer risk in northern China (CR > 10^−6^) but no obvious carcinogenic effects were found to have occurred (HQ < 1) [16]. Another study conducted in eastern China reported that the maximum concentration of the HQ values of TBP, TCEP, and TCPP ranged from 10^−4^ to 10^−3^, and those of the other five OPFRs (TBEP, TCP, TEHP, TPHP, and TDCPP) were all less than 10^−4^ for the age and gender groups of people who chose tap water as their regular drinking water [18]. That study also found that the CR values of the TCEP exposure were 1.1 × 10^−8^–3 × 10^−8^. These results are consistent with our data. Hence, the exposure of OPFRs through the ingestion of tap water by Shanghai residents was not found to pose a potential health risk. Considering that the R*f*D and SFO values were obtained from foreign studies, it is necessary to develop Chinese regulatory values for more appropriate assessments of the carcinogenic and chronic non-carcinogenic risks for local residents. The epidemiological associations between exposure to OPFRs and health effects in humans may deserve attention [43]. For examples, TCIPP has been linked with adverse respiratory outcomes, while TCEP is associated with an increased risk of papillary thyroid cancer in women. A more systematic assessment of multiple health outcomes may be useful in identifying overall health risk but potential hazards on human health and their dose–response relationships are still unclear. In addition, information regarding the joint effects of OPFRs and their coexisting characteristics is lacking.

The Chinese usually consume tap water after boiling. Previous studies have been conducted on the effect of boiling water. Lee et al. (2016) reported that the concentrations of ΣOPFR, TCEP, TCPP, and TBEP in tap water decreased after boiling for 1 h, with the reduction rates ranging from 40% to 60% [25]. Li et al. (2014) found that the concentrations of TBP and TEHP in tap water decreased after boiling, while the levels of TBEP, TPP, and TCPP increased [7]. Ding et al. (2015) reported that the total OPFR concentration in boiled tap water was just slightly increased from 192 ng/L to 212 ng/L [8]. Briefly, most of the OPFR concentrations did not significantly change. The reduction was likely due to the hydrolysis or evaporation of OPFRs during the boiling process, but the mechanism is still unclear. For the purpose of a more conservative assessment, we used un-boiled water samples to assess the ingestion risk.

### 3.5. Risk Management

Until now, several studies have reported the health risk of OPFRs in global drinking water, and we believe that this study could contribute significantly to the knowledge of the OPFR pollution status in the drinking water system of China. The main strength of this study was that our investigation covered all of the water types in the tap water system with a large sample. The data for the OPFR levels in the tap water system were obtained by repeated measures across wet and dry seasons. Nonetheless, the assessment was subject to some limitations and uncertainties.

A main uncertainty in this risk assessment could be the input parameters of the modeling for the OPFR levels in the tap water system. Variability can be due to regional, seasonal, treatment technology, and water pipeline differences. The considerable degree of variability in the environmental concentrations of pollutants was generally found when comparing the results from government monitoring programs and the scientific literature [44]. Another similar source of uncertainty came from information on the frequency and direct ingestion of tap water.

The present study has several limitations. First, some OPFR analogs in the environment were not investigated or calculated for health risk. Second, the exposure assessment did not include information on the possible pathways that could be involved in dermal absorption and indirect ingestion during swimming and/or bathing events, which could lead to underestimations. Third, the generalizability of the study findings could be limited due to the study only being conducted in Pudong New Area, Shanghai. In addition, the water consumption and/or the water flow rate of the sampling sites were not included in the survey, which could also bias the study findings.

The concentrations of TCIPP, TCEP, and TBP, the major OPFRs in the tap water system, were inconsistent with the results of previous relevant studies in China but similar to those in developed countries [4,7,8,18]. This indicates that changes have taken place in the compositional profiles of OPFR pollution. Given the evidence that the global consumption of OPFRs is increasing, this study could provide baselines for future government monitoring programs of China. In addition, it is recommended that a series of policies should be introduced to restrict the usage and production of these compounds in China. Currently, the application of tertiary water treatment-activated carbon filtration in waterworks is a cost–benefit way through which to reduce the threat of OPFRs to tap water safety.

## 4. Conclusions

In this study, ten OPFRs in the tap water of Shanghai were investigated to better understand the occurrence and distribution of OPFRs in daily drinking water. The preliminary exposure and risk of OPFRs were also assessed among various population groups. It was found that TBP, TCIPP, and TCEP were the main OPFR contaminants in tap water due to their relatively higher detected frequencies and concentrations. The tap water intake could be an important source of OPFR exposure. The dose was comparable with that from airborne exposure via particulate matter inhalation. Nevertheless, both non-carcinogenic and carcinogenic risk assessment results revealed very low risks originating from exposure to OPFRs via the direct ingestion of tap water in Shanghai.

## Figures and Tables

**Table 1:** Concentrations of the organophosphate flame retardants in the tap water of Pudong New Area, Shanghai.

Conc. Unit (ng/L)		TBP	TCIPP	TCEP	TEP	TPrP	TPhP	TBEP	TDBPP	TDCPP	TCP	∑_10_OPFRs
2021.12	MIN	8.96	20.9	<LOQ	-	-	-	<LOQ	-	-	-	53.0
(Dry season)	MAX	162	81.4	49.6	-	-	-	12.5	-	-	-	251
*n* = 15	MEAN	45.9	46.3	25.3	-	-	-	2.03	-	-	-	120
	MEDIAN	21.1	42.7	24.5	-	-	-	-	-	-	-	95.0
	D.F.	100%	100%	93%	0%	0%	0%	20%	0%	0%	0%	100%
2022.07	MIN	<LOQ	66.1	17.0	-	-	-	-	-	-	-	83.0
(Wet season)	MAX	31.3	348	61.0	-	-	-	-	-	-	-	425
*n* = 15	MEAN	16.5	167	33.8	-	-	-	-	-	-	-	218
	MEDIAN	18.1	173	33.5	-	-	-	-	-	-	-	223
	D.F.	93%	100%	100%	0%	0%	0%	0%	0%	0%	0%	100%
2023.02	MIN	<LOQ	<LOQ	<LOQ	-	-	<LOQ	-	-	-	-	11.0
(Dry season)	MAX	25.3	137	44.6	-	-	5.47	-	-	-	-	197
*n* = 15	MEAN	11.2	57.4	16.9	-	-	0.36	-	-	-	-	86.0
	MEDIAN	11.7	47.4	18.2	-	-	-	-	-	-	-	79.5
	D.F.	80%	73%	67%	0%	0%	7%	0%	0%	0%	0%	100%
2023.07	MIN	<LOQ	60.4	15.7	-	<LOQ	<LOQ	-	-	<LOQ	<LOQ	93.8
(Wet season)	MAX	8.47	154	100	-	31.1	31.1	-	-	10.7	16.0	259
*n* = 15	MEAN	4.51	95.9	46.3	-	4.90	4.17	-	-	4.27	2.07	158
	MEDIAN	3.71	91.8	44.7	-	-	-	-	-	5.30	-	153
	D.F.	93%	100%	100%	0%	25%	20%	0%	0%	60%	33%	100%

D.F.: detection frequency.

**Table 2:** The heath risk via tap water ingestion for normal- and high-exposure scenarios.

		High Exposure		Normal Exposure	
		MIN ^1^	MAX ^2^	MIN ^3^	MAX ^4^
Hazard quotient (HQ) ^5^	TBP	8.5 × 10^−5^	2.7 × 10^−4^	2.3 × 10^−5^	7.8 × 10^−5^
	TCIPP	4.1 × 10^−4^	1.3 × 10^−3^	1.6 × 10^−4^	5.3 × 10^−4^
	TCEP	2.0 × 10^−4^	6.2 × 10^−4^	8.0 × 10^−5^	2.7 × 10^−4^
Hazard index (HI)	∑OPFRs	6.9 × 10^−4^	2.2 × 10^−3^	2.6 × 10^−4^	8.8 × 10^−4^
Carcinogenic risk (CR) ^6^	TBP	7.7 × 10^−9^	2.4 × 10^−8^	2.1 × 10^−9^	7.0 × 10^−9^
	TCEP	2.7 × 10^−8^	8.7 × 10^−8^	1.1 × 10^−8^	3.7 × 10^−8^

^1^: The 12 to <15 year age group; ^2^: the 6 to <9 month age group; ^3^: the 12 to <15 year age group; ^4^: the 1 to <2 year age group; ^5^: the R*f*D of the TBP, TCIPP, and TCEP was 1 × 10^4^, 1 × 10^4^ and 7 × 10^3^ ng/kg bw/day, respectively [16,17]; and ^6^: the SFO of the TBP and TCEP was 9 × 10^−9^ and 2 × 10^−8^ (ng/kg bw/day)^−1^, respectively [18].

## Data Availability

The data presented in this study are available on request from the corresponding authors.

## Supplementary Materials

The following supporting information can be downloaded at: https://www.mdpi.com/article/10.3390/toxics12100696/s1, Text S1: Sample collection; Text S2: Analytical methods. Figure S1: Material distribution of the transport pipes in tap water; Figure S2: MRM chromatograms of 10 OPFRs and the internal standard (100 ng/L); Figure S3: A map of the monitoring points of drinking water treatment plants and piped water; Figure S4: The compositional profiles of OPFRs in the before- and after-treatment plant water (b-TPW; a-TPW, respectively); tap water supplied directly by pipes (p-TW); tap water supplied through water storage tanks (s-TW); and tap water (TW); Figure S5: The ADD of the OPFRs in males and females under normal- and high-exposure scenarios; Table S1: Linear equations, correlation coefficients, detection limits, and quantitative limits of the 10 OPFRs; Table S2: Age-specific daily direct drinking water ingestion rates; Table S3: Gender-specific daily direct drinking water ingestion rates; Table S4: The R*f*D (ng/kg bw/day) and SFO values ((ng/kg bw/day)^−1^) of the OPFRs; Table S5: Comparisons of the OPFR detection rates (%) and concentrations (ng/L) among previous studies; and Table S6: The correlations of TBP, TCIPP, and TCEP.
