# Supplementary material for: Occurrence and Distribution of Organophosphate Flame Retardants in Tap Water System—Implications for Human Exposure from Shanghai, China
Source: Toxics. 2024 Sep 26;12(10):696. doi: 10.3390/toxics12100696 (PMC11510868; doi:10.3390/toxics12100696)
Supplement: Supplementary file 1 [file toxics-12-00696-s001.zip › toxics-3162709-supplementary.pdf]

Article

Occurrence and Distribution of Organophosphate Flame Retardants in Tap Water System—Implications for Human Exposure from Shanghai, China

Yuan-Shen Zhu [1,2], Lei Zheng [1,2], Wei-Wei Zheng [3,4], Rong Zheng [1,2], Ya-Juan Wang [1,2], Bing-Qing Hu [1,2], Min-Juan Yang [1,2,*] and Yi-Jing Zhao [1,2,*]

1 Center for Disease Control and Prevention, Pudong New Area, Shanghai 200136, China; 18211020127@fudan.edu.cn (Y.-S.Z.); zhengcdc@163.com (L.Z.); rzheng@pdcdc.sh.cn (R.Z.); yjwang@pdcdc.sh.cn (Y.-J.W.); bqhu@pdcdc.sh.cn (B.-Q.H.)
2 Fudan University Pudong Institute of Preventive Medicine, Shanghai 200136, China
3 Key Laboratory of the Public Health Safety, Ministry of Education, Department of Environmental Health, School of Public Health, Fudan University, Shanghai 200032, China; weiweizheng@fudan.edu.cn
4 Center for Water and Health, School of Public Health, Fudan University, Shanghai 200032, China
* Correspondence: mjyang@pdcdc.sh.cn (M.-J.Y.); yjzhao@pdcdc.sh.cn (Y.-J.Z.)

Contents:

Text S1 Sample collection

Sixty tap water samples were periodically collected from fifteen monitoring points of drinking water in Pudong New Area between November 2021 and July 2023, representing drinking water for more than 5 million people in Shanghai. Fourteen before-treatment plant water (b-TPW, also as raw water) samples and thirty-one after-treatment plant water (a-TPW, also as finished water) samples were collected from seven waterworks in Pudong New Area. After opening the tap, outflow in the first 1 min was discarded. We used a solvent-cleaned glass bottle to collect 500mL of water for each sample, and sealed it with Parafilm™ M Wrapping Film, purchased from Bemis Company INC. (Sheboygan Falls, WI, USA). All samples were stored at 4°C until analysis. The analysis was conducted within 48h after sampling.

Sequence	Season	Tap water (n)	After-treatment plant water (n)	Before-treatment plant water (n)	Total (n)
2021.12	dry	15	8	-	23
2022.07	wet	15	8	-	23
2023.02	dry	15	8	7	30
2023.07	wet	15	8	7	30

In this study, 23 monitoring points consisted of 8 water treatment plants and 15 tap waters (TW), 8 of 15 supplied directly by pipes (p-TW) and 7 of 15 supplied through water storage tanks (s-TW):

Waterworks	Area	Before and after-treatment plant water	Tap water supplied directly by pipes	Tap water supplied through water storage tanks
HN	surrounding	1	1	1
HT	surrounding	1	1	1
JH	central	1	1	1
JJQ	central	1	1	1
LJ	central	1	2	1
LJZ	central	1	1	1
LQ	central	1	0	1
PD	central	1	1	0

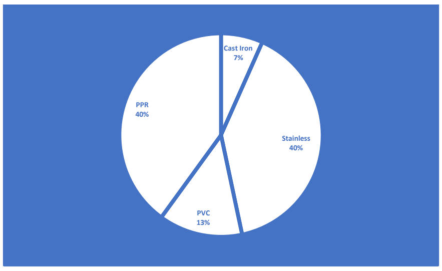

Figure S1. Material distribution of transport pipes in tap water.

Text S2 Analytical methods

Each water sample was mixed thoroughly, and 50mL was spiked with TPhP-d_{15} as internal standard and filtered with polyethersulfone (PES) filters (0.22μm, Waters, USA) which was reported for dissolved phase of OPFRs. The filtration was repeated triple. The third filtrate (5mL) was reserved and extracted using an on-line solid-phase extraction (SPE) method with cartridge (Sep-Pak C8, 500 mg, 3cc: Waters, Milford, MA, USA). The mobile phase consisted of HPLC grade water (A), HPLC grade water with 0.5% formic acid (B), acetonitrile and methanol (9:1) with 0.1% formic acid (C), and methanol (MeOH) and acetone and hexane (1:1:1) (D). The gradient elution program was as follows (with reference to A, B, C, D):

Time (min)	Flow rate (mL/min)	Mobile phase
0	0.5	90%A+10%C
0.5	0.5	95%B+5%C
3.8	0.01	95%B+5%C
4.1	1.5	100%A
5.6	1.5	100%D
7.6 & 10	1.5	5%A+95%C
10.5 & 13	1.0	90%A+10%C

In brief, a 5μL sample was injected into a Waters BEH C18 column (2.1mm × 50mm, 1.7μm). The mobile phase consisted of HPLC grade water with 0.1% formic acid (A1) and acetonitrile (B1) was used for the separation of analytes at a flow rate of 0.35mL/min. The gradient was as follows (with reference to A1, B1):

Time (min)	Mobile phase
0	70%A1+30%B1
3.8	70%A1+30%B1
4.1	70%A1+30%B1
10	5%A1+95%B1
10.3	70%A1+30%B1
13	70%A1+30%B1

The mass spectra were performed in multiple reaction mode (MRM) using an electrospray ionization (ESI) in positive ion mode. The quantitative ion parameters are as follows:

Compounds	Retention time (min)	Precursor ion (m/z)	Product ion (m/z)	Collision energy (V)	Cone voltage (V)
TEP	6.12	182.9	98.7*	18	26
			126.8	18	26
TCEP	6.74	284.9	98.8*	20	34
			124.9	20	34
TPrP	7.45	225.0	98.8*	22	24
			141.0	22	24
TCIPP	7.71	326.9	98.8*	26	32
			174.9	26	32
TDCPP	8.56	432.8	98.8*	22	22
			210.4	16	28
TPhP	8.80	326.9	76.9*	38	60
			151.9	38	60
TDBPP	8.82	698.4	98.7*	44	34
			118.3	34	32
TBP	8.92	267.1	98.8*	16	20
			155.0	16	20
TBEP	9.29	399.1	100.9	16	36
			199.0*	16	36
TCP	9.81	369.1	90.9*	42	58
			165.2	42	58
TPhP-d$_{15}$	8.79	342.0	81.8*	40	64
			159.7	40	64

*: quantification ion.

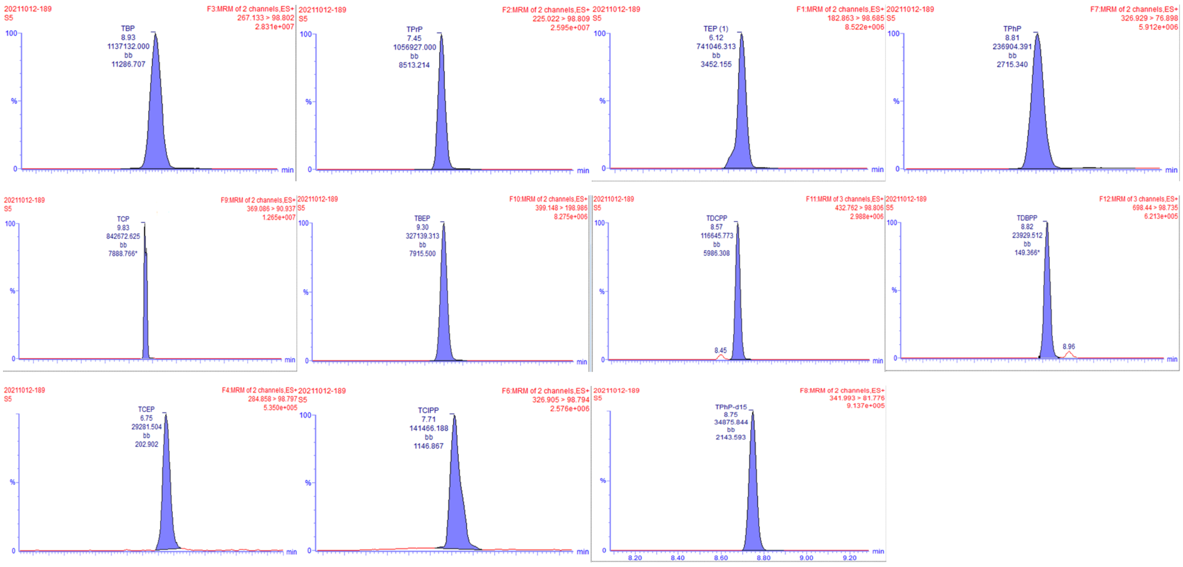

Figure S2. MRM chromatograms of 10 OPFRs and internal standard (100ng/L).

Table S1. Linear equations, correlation coefficients, detection limits and quantitative limits of 10 OPFRs.

Compounds	Linear equations	Correlation coefficients	Detection limits (ng/L)	Quantitative limits (ng/L)
TBP	$y = 0.3155x + 0.1827$	0.9960	1.1	3.7
TPrP	$y = 0.3063x + 0.3411$	0.9998	0.6	1.9
TEP	$y = 0.1971x + 0.1943$	0.9997	1.4	4.6
TPhP	$y = 0.0672x + 0.1624$	0.9952	1.2	3.9
TCP	$y = 0.2388x - 0.5402$	0.9950	0.6	2.0
TBEP	$y = 0.0918x - 0.2632$	0.9951	1.0	3.3
TDCPP	$y = 0.0328x + 0.0452$	0.9989	1.0	3.2
TDBPP	$y = 0.0067x - 0.0186$	0.9962	1.5	5.0
TCEP	$y = 0.0082x + 0.0734$	0.9999	2.4	7.8
TCIPP	$y = 0.0369x + 1.2651$	0.9991	5.5	18.2

Table S2. Age-specific daily direct drinking water ingestion rates[a].

Age groups	Mean (mL)	P_{95} (mL)	Body weight[b] (kg)
Birth to <3 months	170	693	7.5
3 to <6 months	378	960	8.6
6 to <9 months	416	1500	10.6
9 to <12 months	701	1313	10.8
1 to <2 years	796	1330	12.2
2 to <3 years	661	1400	14.2
3 to <4 years	570	900	16.4
4 to <5 years	566	1000	18.1
5 to <6 years	617	1014	20.6
6 to <9 years	918	2400	28.1
9 to <12 years	1053	2450	39.5
12 to <15 years	1009	2300	51.4
15 to <18 years	1159	2700	58.1
18 to <45 years	1776	4125	61
45 to <60 years	1882	4500	64
60 to <80 years	1733	4250	62
≥80 years	1628	5000	57.4

([a]: Data from Exposure Factors Handbook of Chinese Population for Shanghai residents[1-3], [b]: Mean value)

Table S3. Gender-specific daily direct drinking water ingestion rates[c].

Gender groups	Mean (mL)	P_{95} (mL)	Body weight[d] (kg)
Male	2745	8400	68.7
Female	2168	4450	58.1

([c]: Data from Exposure Factors Handbook of Chinese Population for Shanghai adult residents[1], [d]: Mean value)

Table S4. R*f*D (ng/kg *bw*/day) and SFO values ((ng/kg *bw*/day)$^{-1}$) of OPFRs.

OPFRs	R*f*D	SFO
TBP	1×10^{4}	9×10^{-9}
TCEP	7×10^{3}	2×10^{-8}
TCIPP	1×10^{4}	-

(Data from US-EPA (2017), Li et al. (2019) and Zhang et al. (2022) [4-6])

Table S5. Comparisons of OPFRs detection rates (%) and concentrations (ng/L) among previous studies.

Study	Sample size	Period	ΣOPFRs		TBP		TCIPP		TCEP		Target OPFRs
			D.F.	Average (Range)	D.F.	Average (Range)	D.F.	Average (Range)	D.F.	Average (Range)	
This study	60	2021-2023	100	124[e] 138[f] (11.0-425)	90	14.9[e] 19.5[f] (ND-162)	78	69.6[e] 91.7[f] (ND-348)	83	29.0[e] 30.6[f] (ND-100)	TBP,TCIPP,TCEP, TEP,TPrP,TPhP,TBEP, TDBPP,TDCPP,TCP
Zhang et al., China[6]	47	—	—	95.7[f] (9.3-225)	96	19.6[f] (0.1-42.3)	—	—	99	18.7[f] (0.3-44.1)	TBEP,TBP,TCP, TEHP,TPHP,TDCPP, TCEP,TBPP,TCPP
Zhang et al., China[7]	25	—	100	125[e] (46.8-251)	100	14.9[e] (4.8-15.6)	—	—	100	43.7[e] (4.9-107)	TCPP,TCEP,TnBP, TDCPP,TPTP,TPhP, EHDPP,TMTP,TEHP
Kim et al., USA[8]	58	2016	100	41.6[f] (3.02-366)	40	2.47[f] (ND-133)	91	11.6[f] (ND-67.1)	9	0.45[f] (ND-17.4)	TMPP,TEP,TPhP,TPP, TBOEP,TCEP,TCIPP, TDBPP,TDCIPP,TEHP, EHDPP,PBDPP,TBP
Ding et al., China[9]	21	2014	—	192[e] (123-338)	100	9.5[e] (3.9-76.3)	—	—	100	48.5[e] (28.5-139)	TEP,TnBP,TnPP, TCEP,TBEP,TPhP, TCPP,TDCIPP,TCrP
Lee et al., Korea[10]	75	2014	—	34.9[f]	—	4.29[f]	—	—	—	25.3[f]	TPP,TCPP,TEP,EHDPP, TBP,TCEP,TDCPP, TBEP,TEHP,TCP
Li et al., China[11]	39	2012	—	165[f] (85.1-325)	100	7.48[f]	—	—	100	12.5[f]	TDCPP,TDBPP,TEHP, TBP,TBEP,EHDPP, TPP,TCPP,TCEP
Rodil et al., Spain[12]	28	2008	—	82[e]	>60	32[e] (11-148)	—	—	>60	5[e]	TCEP,TEP,TCPP,TnBP

(TBP is summated concentration of TiBP and TnBP, D.F.: Detection frequency, ND: No detected, [e]: Median value, [f]: Mean value)

Table S6. Correlations of TBP, TCIPP and TCEP.

		TBP	TCIPP	TCEP
ALL	**TBP**	1.000	0.166	**0.204***
(n=106)	**TCIPP**	0.166	1.000	**0.699****
	**TCEP**	**0.204***	**0.699****	1.000
TAP WATER	**TBP**	1.000	0.058	-0.001
(n=60)	**TCIPP**	0.058	1.000	**0.721****
	**TCEP**	-0.001	**0.721****	1.000
b-TW	**TBP**	1.000	0.109	**0.538***
(n=14)	**TCIPP**	0.109	1.000	**0.714****
	**TCEP**	**0.538***	**0.714****	1.000
a-TW	**TBP**	1.000	0.310	0.327
(n=32)	**TCIPP**	0.310	1.000	**0.542****
	**TCEP**	0.327	**0.542****	1.000

(*: Correlation is significant at the 0.05 level (2-tailed), **: Correlation is significant at the 0.01 level (2-tailed))

toxics

FIGURE

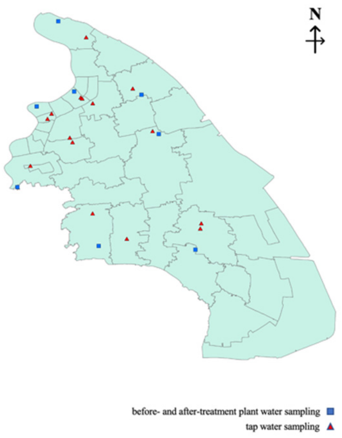

Figure S3. A map of monitoring points of drinking water treatment plants and piped water.

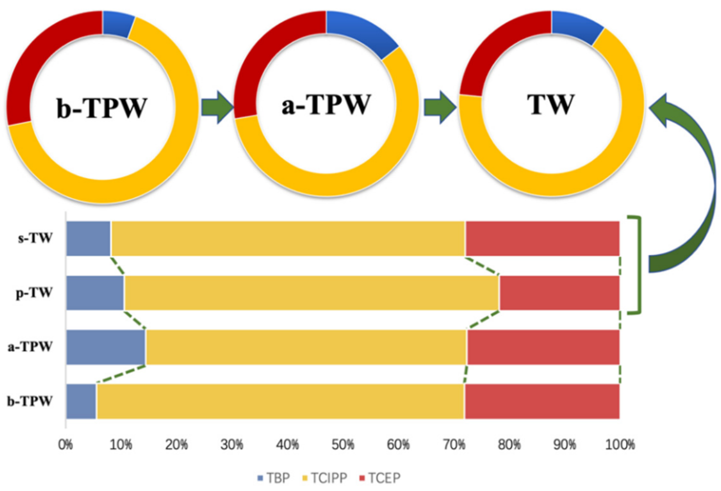

Figure S4. Compositional Profiles of OPFRs in before and after-treatment plant water (b-TPW; a-TPW), tap water supplied directly by pipes (p-TW), tap water supplied through water storage tanks (s-TW) and tap water (TW).

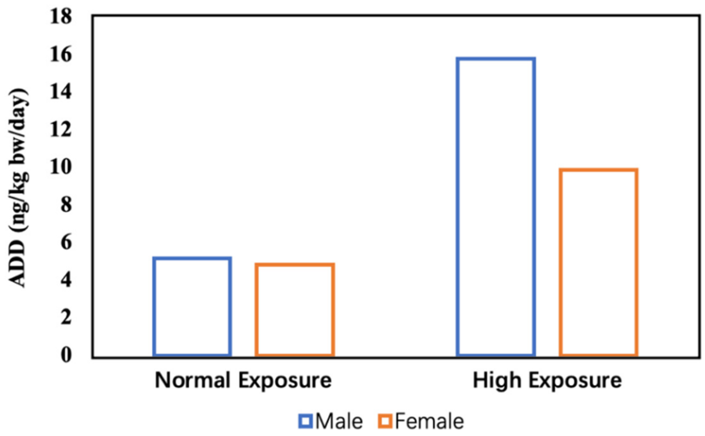

Figure S5. The ADD of OPFRs in males and females under normal- and high exposure scenarios.

References

1. MEPC. *Exposure Factors Handbook of Chinese Population for Shanghai adult residents*; China Environmental Science Press: Beijing, 2013.
2. MEPC. *Exposure Factors Handbook of Chinese Population for Shanghai residents (0-5 years old)*; China Environmental Science Press: Beijing, 2016.
3. MEPC. *xposure Factors Handbook of Chinese Population for Shanghai residents (6-17 years old)*; China Environmental Science Press: Beijing, 2016.
4. USEPA. Mid Atlantic risk assessment, Regional Screening Levels (RSLs) - Generic Tables: Washington, D.C, 2017. (http://www.epa.gov/region9/superfund/prg) (accessed September, 2024).
5. Li, J.; He, J.; Li, Y.; Liu, Y.; Li, W.; Wu, N.; Zhang, L.; Zhang, Y.; Niu, Z. Assessing the threats of organophosphate esters (flame retardants and plasticizers) to drinking water safety based on USEPA oral reference dose (RfD) and oral cancer slope factor (SFO). Water Res 2019, 154, 84-93, doi:10.1016/j.watres.2019.01.035.
6. Zhang, Q.; Li, J.; Lin, S.; Ying, Z.; Hu, S.; Wang, Y.; Mo, X. Organophosphate flame retardants in Hangzhou tap water system: Occurrence, distribution, and exposure risk assessment. *Sci. Total Environ.* **2022**, *849*, 157644, doi: 10.1016/j.scitotenv.2022.157644.
7. Zhang, S.; Li, Y.; Yang, C.; Meng, X.Z.; Zheng, H.; Gao, Y.; Cai, M. Application of Hi-throat/Hi-volume SPE technique in analyzing occurrence, influencing factors and human health risk of organophosphate esters (OPEs) in drinking water of China. *J. Environ. Manage.* **2021**, *291*, 112714, doi: 10.1016/j.jenvman.2021.112714.
8. Kim, U.J.; Kannan, K. Occurrence and Distribution of Organophosphate Flame Retardants/Plasticizers in Surface Waters, Tap Water, and Rainwater: Implications for Human Exposure. *Environ. Sci. Technol.* **2018**, *52*, 5625-5633, doi: 10.1021/acs.est.8b00727.
9. Ding, J.; Shen, X.; Liu, W.; Covaci, A.; Yang, F. Occurrence and risk assessment of organophosphate esters in drinking water from Eastern China. *Sci. Total Environ.* **2015**, *538*, 959-965, doi: 10.1016/j.scitotenv.2015.08.101.
10. Lee, S.; Jeong, W.; Kannan, K.; Moon, H.B. Occurrence and exposure assessment of organophosphate flame retardants (OPFRs) through the consumption of drinking water in Korea. *Water Res.* **2016**, *103*, 182-188, doi: 10.1016/j.watres.2016.07.034.
11. Li, J.; Yu, N.; Zhang, B.; Jin, L.; Li, M.; Hu, M.; Zhang, X.; Wei, S.; Yu, H. Occurrence of organophosphate flame retardants in drinking water from China. *Water Res.* **2014**, *54*, 53-61, doi: 10.1016/j.watres.2014.01.031.
12. Rodil, R.; Quintana, J.B.; Concha-Grana, E.; Lopez-Mahia, P.; Muniategui-Lorenzo, S.; Prada-Rodriguez, D. Emerging pollutants in sewage, surface and drinking water in Galicia (NW Spain). *Chemosphere* **2012**, *86*, 1040-1049, doi: 10.1016/j.chemosphere.2011.11.053.
